# A DNA methylation profile of long non-coding RNAs can predict OS in prostate cancer

**DOI:** 10.1080/21655979.2021.1945991

**Published:** 2021-07-08

**Authors:** Wei Cheng, Jie Cao, Yong xia, Xin Lei, Lili Wu, Liang Shi

**Affiliations:** a Department of Neurology, Suizhou Hospital, Hubei University of Medicine, Suizhou, China; b Department of Tanslational Medicine Laboratory, the First Affiliated Hospital of Wenzhou Medical University, Wenzhou, China; c Department of Clinical Medical Laboratory, Peking University Shenzhen Hospital, Shenzhen, China; d Department of Clinical Transfusion, The Eighth Affiliated Hospital, Sun Yat-sen University, Shenzhen, China; e Department of Laboratory Medicine, The Eighth Affiliated Hospital, Sun Yat-sen University, Shenzhen, China

**Keywords:** Prostate cancer, lncRNAs, methylation, prognostic

## Abstract

Prostate cancer (PCa) is the most common male reproductive tract malignant tumor, accurate evaluation of PCa characterization and prognostic prediction at diagnosis are vital for the effective administration of the disease, especially at the molecular level. In this study, 48 CpG sites with differential methylation associated with overall survival (OS) were screened out between PCa and normal adjacent tissues. 16 CpG sites were selected by the least absolute shrinkage and selection operator (LASSO) and the risk score formula for methylated-based classifier was established. For 16-lncRNAs-CpG-classifier, the area under the curve (AUC) were 0.890, 0.917, and 0.932 at 3 years, 5 years and 7 years, respectively. Kaplan–Meier curves indicated that patients with high-risk scores had worse OS than those with low-risk scores. Prognostic methylation model of lncRNAs was identified from the whole genome in patients with PCa. This novel finding provides a novel insight for screening biomarkers of a prognosis for PCa.

## Graphical abstract

**Figure:**
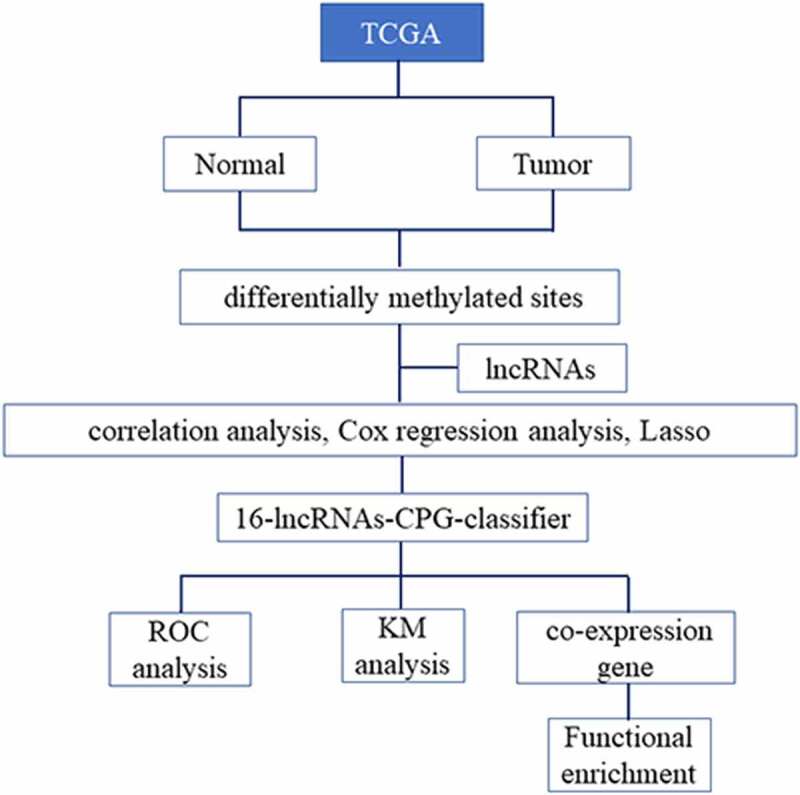

## Introduction

Prostate cancer (PCa) is the most frequent male reproductive tract malignant tumor, which is the fifth cause of male cancer death in the world [1]. Along with aging population structure and changes in diet, coupled with male hormone use undeserved, the incidence of PCa is rising globally. Although discovering and treating of PCa before symptoms happen may not promote their health or make them live longer due to usually growing very slowly, accurate evaluation of PCa characterization and prognostic prediction at diagnosis is momentous for the useful management of the cancer, especially at the molecular level [2].

Over the past decade, the rapid progress of genomic techniques and their application for deciphering the genome of cancer have provided new diagnostic value and prognostic assessment for PCa patients. DNA methylation is formed by the covalent addition of methyl groups and DNA bases, typically the CpG dinucleotide cytosine, which lead to reversible alterations in gene expression of key tumor suppressor genes without permanent changes in DNA sequence in tumorigenesis [3]. Furthermore, DNA methylation as a biomarker for cancers describes stable changes, which could improve individual PCa risk assessment and prognostic prediction [4].

Long non-coding RNAs (lncRNAs) are crucial to regulate the gene expression and are associated with many biological processes, including tumorigenesis in mammals. Likewise, lncRNAs are frequently dysregulated in cancers, including PCa [5]. Furthermore, lncRNAs with aberrant methylation patterns significantly affect the occurrence and development of tumors. For example, it has been revealed that deregulation of the DNMT1-related lncRNAs conduced to aberrant gene expression and DNA methylation levels [6]. However, little is known about the DNA methylation pattern of lncRNAs in PCa [7].

In this study, we explored the methylation sites of lncRNAs between PCa and para-carcinoma tissues to screen out differential methylation sites. Based on correlation analysis and LASSO regression analysis, a methylated classifier of lncRNAs was developed for assessing the prognosis of PCa. Furthermore, we also predicted the prognosis value of methylation-based classifiers, which could guide individualized clinical treatment for PCa patients.

## Methods

### Patient datasets

The DNA methylation data, RNA-seq data and clinical information of PCa patients were acquired from The Cancer Genome Atlas (TCGA) database (https://portal.gdc.cancer.gov/) [8]. DNA methylation profile was performed by Infinium HumanMethylation450 BeadChip. The RNA-seq was performed on IlluminaHiSeq RNA-seq platform [9]. The annotation file of lncRNA was acquired from GENCODE (https://www.gencodegenes.org/) [10]. All datasets were publicly available and this research met the publication guidelines.

### CpG sites of lncRNAs

The methylation of CpG sites, which were reported as the beta-value, ranged from 0 (no methylation) to 1 (complete methylation). The methylation beta-values were normalized in R software using ‘minfi’ package [11]. Based on the annotation of TCGA, CpG sites in 2kb upstream of lncRNAs transcriptional start site (TSS) were filtered from 485,577 sites in HumanMethylation450 BeadChip. Differential methylation sites of lncRNA between PCa and para-carcinoma tissues were screened using ‘minfi’ package, and the q-value was less than 0.05 to be considered statistically significant. The T-test was applied to calculate the differential beta-values of CpG sites between PCa and para-carcinoma tissues, and the differential beta-values were more than 0.1 to be considered meaningful. Then, correlation analysis was performed to screen CpG sites, where the methylated levels were negatively associated with lncRNA expression levels, and the p-value < 0.05 was considered statistically significant.

### Methylation-based classifier for overall survival (OS)

Univariate Cox regression analysis was performed to calculate the correlation between methylation level of CpG sites filtered in the above step and patient’s OS, and the p-value < 0.05 was considered in this study. Next, a Least Absolute Shrinkage and Selection Operator (LASSO) regression model was performed to screen the most useful prediction CpG sites, and a methylation-based classifier was constructed for predicting OS based on the sum of CpG site methylation levels weighted by the coefficients [12]. LASSO regression is a variable selection and shrinkage method for the regression models to identify the variables and relevant regression coefficients, which are established to minimize the prediction error.

### Predictive and prognostic analysis of methylation-based classifier

Based on the methylation-based classifier, the risk scores of patients were calculated, and the predictive effect of the classifier for OS was assessed by time-dependent receiver operating characteristic (ROC) analysis based on the risk score in R software using ‘timeROC’ package [13]. The area under the curve (AUC) of ROC indicated the predictive or prognostic accuracy. Based on the median of the classifier risk score, patients were separated into high-risk group and low-risk group, and the Kaplan–Meier method was performed to estimate survival for patients, the p-value was less than 0.05 to be considered statistically significant.

### Co-expression gene of lncRNAs and functional enrichment analysis

The co-expression genes of lncRNAs were identified to use MEM (Multi-Experiment Matrix). MEM is a similarity search of gene expression across numerous datasets [14]. The main feature of MEM is that it collects large microarray datasets. It utilizes rank aggregation to integrate information from different datasets into a single global sorting and simultaneously estimates statistical significance. The function enrichment analysis of co-expression genes was performed through the DAVID Bioinformatics Resources (https://david.ncifcrf.gov/) [15]. The FDR of enrichment terms was less than 0.05 to be considered statistically significant, and the enrichment results were visualized by the ‘ggplot2’ package in R software.

### Statistical analysis

Continuous or categorical variables were compared between the two groups by the t-test (normal distribution) or *χ^2^* test, respectively. *P* < 0.05 or *Q* < 0.05 was considered statistically significant. Differential *β*-value > 0.1 between two groups was considered statistically significant. FDR < 0.05 was considered significant.

## Results

In this study, we aimed to develop a prognosis methylation model of lncRNAs to predict the prognosis of PCa patients. We investigated the differential methylation sites of lncRNAs between PCa and para-carcinoma tissues. Based on the correlation analysis and LASSO regression analysis, the methylation prognosis model of lncRNAs was built to predict the prognosis of PCa. In addition, the prognosis value of the methylation-based classifier was assessed, which indicated that this prognosis model could better predict the outcome of PCa patients.

### Characteristics of patient datasets

There were 500 patients of PCa in TCGA, and 498 patients had both the DNA methylation data and survival data. The clinical characteristics of the 498 patients are shown in the Table 1. The methylation data were from 548 samples, including 498 PCa samples, and 50 normal adjacent tissues. The RNA-seq data were 547 samples, and 530 samples had both RNA-seq data and methylation data.

**Table 1.** Clinical characteristics of prostate cancer patients

Clinicopathological variables	n = 498
Age	
< 60 years	203 (40.8%)
≥ 60 years	295 (59.2%)
Subtype	
Acinar Type	483 (97.0%)
Other	15 (3.0%)
Gleason score	
5–7	293 (58.8%)
8–10	205 (41.2%)
Tumor size	
T1 + T2	188 (37.8%)
T3 + T4	303 (60.8%)
Recurrence	91 (18.3%)
Death	10 (2.0%)

### Differential methylation of CpG sites and methylation-based classifier

**Figure 1.**
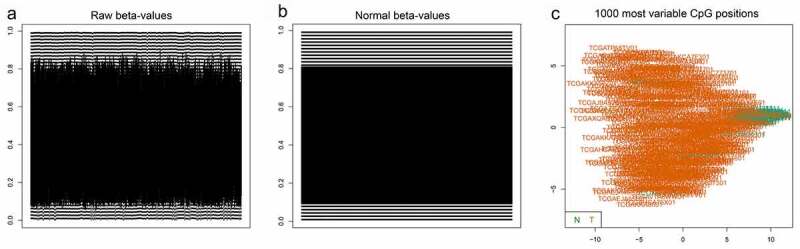
(a) The raw data of beta-values in methylated CpG site.
The normalized beta-values of methylation of CpG site.One thousand most variable CpG sites were screened using ‘minfi’package in R software

**Figure 2.**
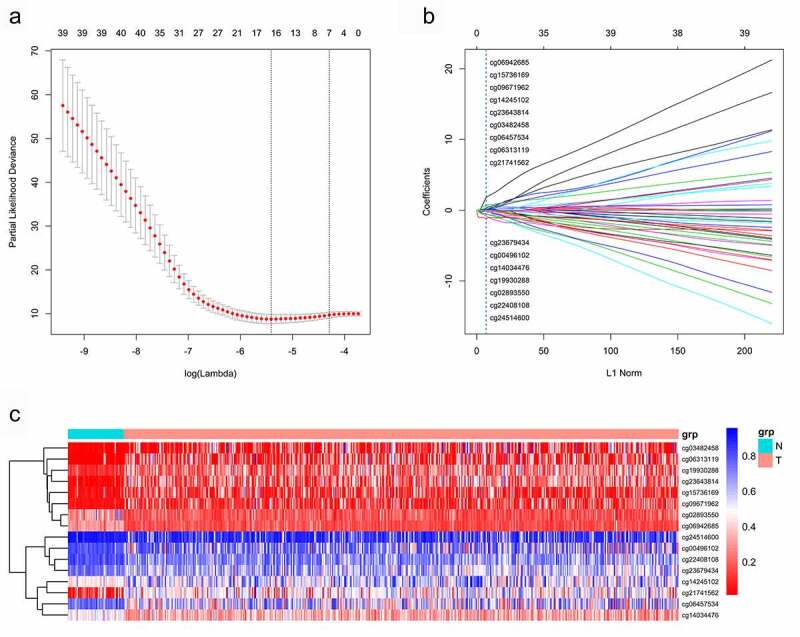
A CpG sites were selected in LASSO analysis.B LASSO coefficient profiles of the CpG sites.C Hierarchical clustering by differential levels in methylated CpG sites

**Figure 3.**
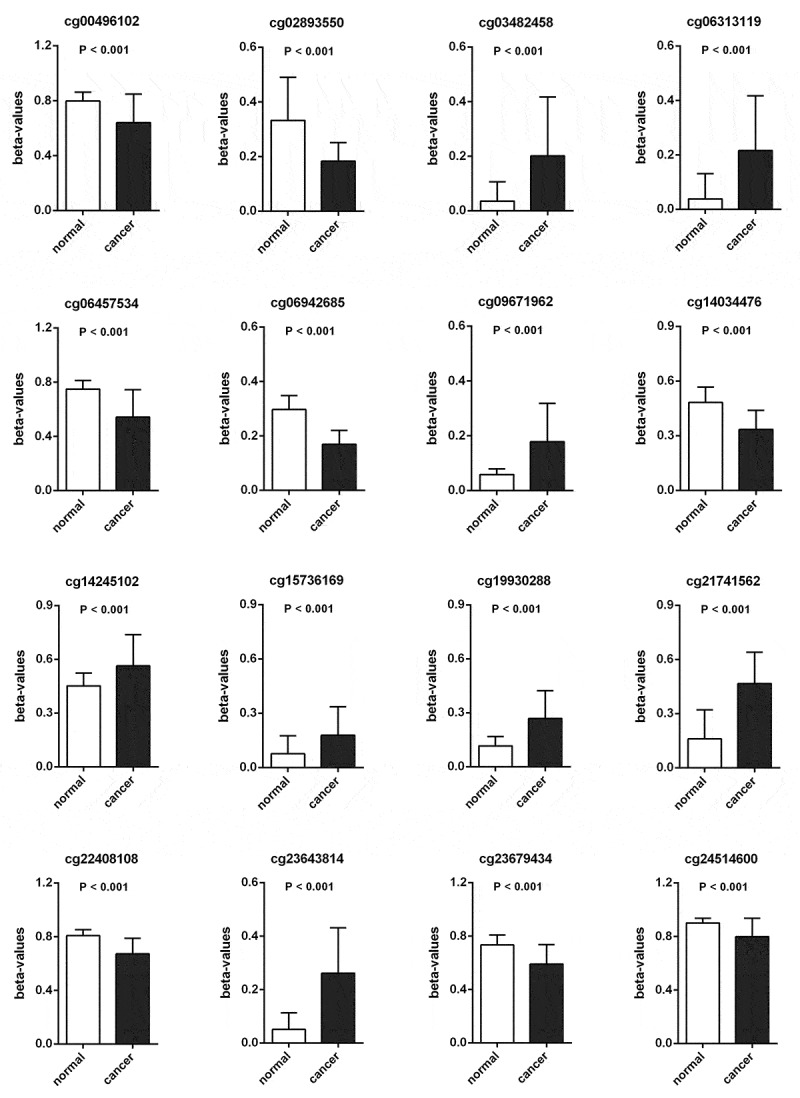
The methylated levels of 8 CpG sites were up-regulated and 8 CpG sites were down-regulated in PCa compared with para-carcinoma tissues

Methylation beta-values were normalized by ‘minfi’ package in R software (Figure 1). Eleven thousand two hundred and fifty-nine CpG sites situated within 2kb upstream of lncRNA TSS (excluding CpG sites on the X and Y chromosomes) were screened out based on the annotation of HumanMethylation450 BeadChip by TCGA. There were 6470 CpG sites with differential methylation levels between PCa and para-carcinoma tissues, which were screened out using ‘minfi’ package, and 1556 CpG sites had different beta-values, which were more than 0.1. Among the 1556 CpG sites, the 484 CpG sites methylation levels were found to be negatively associated with the lncRNAs expression levels. Based on univariate Cox regression analysis, 48 CpG sites were significantly associated with OS. In order to develop methylation-based classifier for predicting OS, the LASSO regression was performed using the methylated data of 48 CpG sites. Ultimately, 16 CpG sites were screened out using LASSO regression analysis (Figure 2a, b). The risk score formula for the methylation-based classifier was established as follows: −0.074 × beta_cg00496102 – 0.497 × beta_cg02893550 + 0.086 × beta_cg03482458 + 0.057 × beta_cg06313119 + 0.068 × beta_cg06457534 + 1.287 × beta_cg06942685 + 0.309 × beta_cg09671962 – 0.175 × beta_cg14034476 + 0.138 × beta_cg14245102 + 0.553 × beta_cg15736169 – 0.484 × beta_cg19930288 + 0.021 × beta_cg21741562 – 0.661 × beta_cg22408108 + 0.122 × beta_cg23643814 – 0.022 × beta_cg23679434 – 1.103 × eta_cg24514600. Table 2 shows the features of the 16 CpG sites, which were selected by LASSO. Compared with para-carcinoma, the methylated levels of eight CpG sites were up-regulated and eight CpG sites were down-regulated in PCa (Figure 3). Unsupervised hierarchical clustering analysis showed that methylated data of the 16 CpG sites could clearly discriminate between PCa and para-carcinoma samples (Figure 2c).

**Table 2.** Characteristics of CpG sites selected by LASSO

CG_ID	Gene_Symbol	CG_Chromosome_location	Position_to_TSS	CGI_Coordinate	Feature_Type
cg00496102	RP5-1159O4.1	chr7: 7,565,607–7,565,608	TSS1500	chr7:7,566,741–7,567,392	N_Shore
cg02893550	CTD-2555A7.2	chr16: 89,053,010–89,053,011	TSS200	chr16:89,052,898–89,053,256	Island
cg03482458	GTSE1-AS1	chr22: 46,296,314–46,296,315	TSS1500	chr22:46,296,313–46,297,452	Island
cg06313119	FGF14-AS2	chr13: 102,394,386–102,394,387	TSS1500	chr13:102,394,330–102,394,876	Island
cg06457534	RP11-201M22.1	chr11: 91,803,313–91,803,314	TSS200	chr11:92,224,469–92,226,866	
cg06942685	AC006116.21	chr19: 56,368,063–56,368,064	TSS200	chr19:56,368,048–56,368,626	Island
cg09671962	LINC01122	chr2: 58,428,328–58,428,329	TSS200	chr2:58,428,308–58,428,977	Island
cg14034476	AC005786.5	chr19: 3,556,720–3,556,721	TSS1500	chr19:3,556,964–3,558,295	N_Shore
cg14245102	MEG3	chr14: 100,826,630–100,826,631	TSS1500	chr14:100,826,526–100,826,764	Island
cg15736169	LINC00403	chr13: 112,106,711–112,106,712	TSS1500	chr13:112,106,551–112,106,799	Island
cg19930288	WWC2-AS2	chr4: 183,100,149–183,100,150	TSS1500	chr4:183,097,677–183,100,226	Island
cg21741562	LINC00506	chr3: 87,089,237–87,089,238	TSS200	chr3:87,089,075–87,089,396	Island
cg22408108	AC091801.1	chr7: 3,175,654–3,175,655	TSS1500	chr7:3,300,722–3,302,101	
cg23643814	RP11-74E22.8	chr17: 2,724,291–2,724,292	TSS1500	chr17:2,724,007–2,725,008	Island
cg23679434	CTB-83J4.1	chr19: 54,224,165–54,224,166	TSS1500	chr19:54,207,238–54,207,507	
cg24514600	PVT1	chr8: 127,793,168–127,793,169	TSS1500	chr8:127,793,835–127,794,653	N_Shore

CGI, CpG island.

### Predictive and prognosis value of the methylation-based classifier

**Figure 4.**
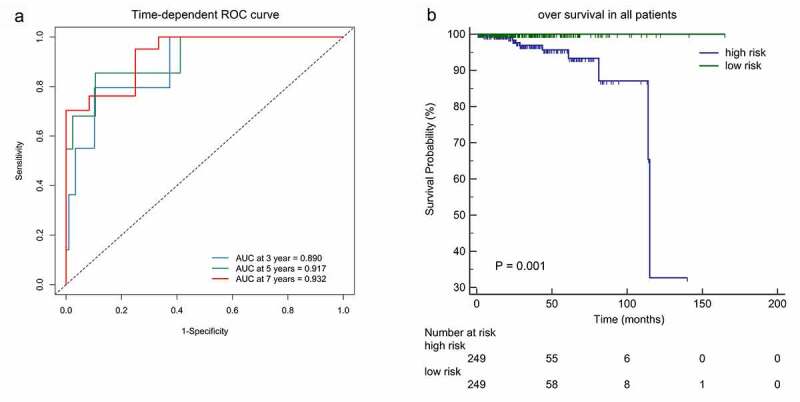
(a)Time-dependent ROC analysis was carried out to estimate the predictive effect for OS at varied follow-up periods.(b) SKaplan–Meier analysis was used to estimate the OS in patients. The patients were divided into high-risk group or low-risk group based on the median cutoff point of risk score

**Figure 5.**
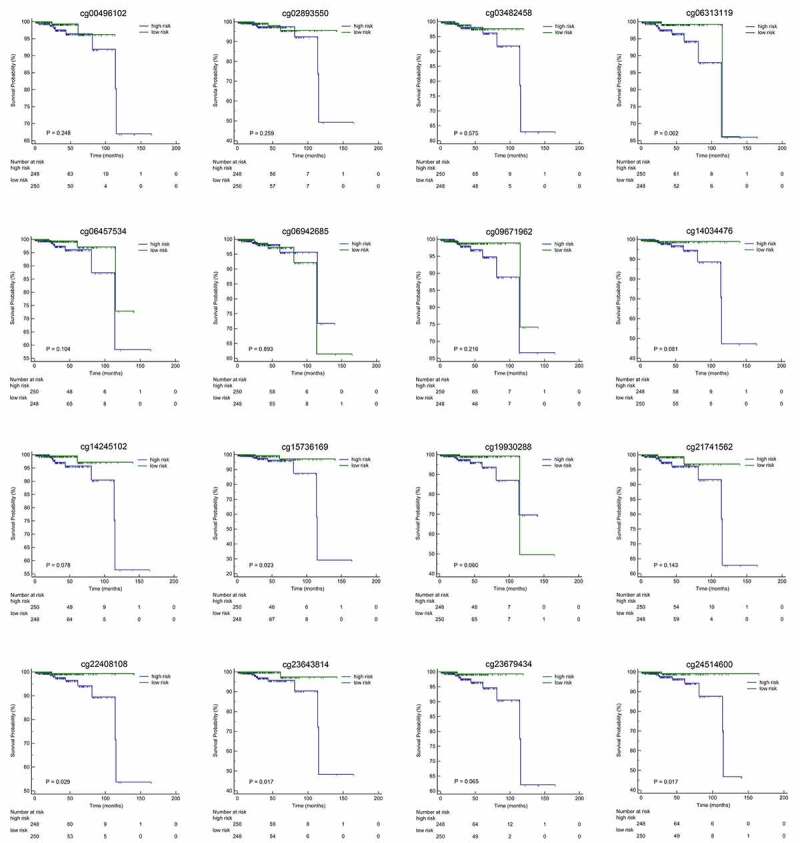
Kaplan-Meier analyses with high-risk or low-risk score of methylation beta-values of single CpG site in the classifier

We calculated the risk score of each patient using a methylation-based classifier and used time-dependent ROC to evaluate the precision of the methylation-based classifier. Time-dependent ROC curve analysis showed that the classifier had a good predictive accuracy at different follow-up periods (AUC at 3 years = 0.890; AUC at 5 years = 0.917; AUC at 7 years = 0.932; Figure 4a). All patients were separated into high-risk or low-risk groups based on the median cutoff point of the risk scores. Kaplan-Meier's analysis indicated that patients in the high-risk group had worse OS than those in the low-risk group (Figure 4b). Kaplan–Meier analysis of a single CpG site in the classifier are also shown in Figure 5.

### Identification lncRNA co-expression gene and functional evaluation

**Figure 6.**
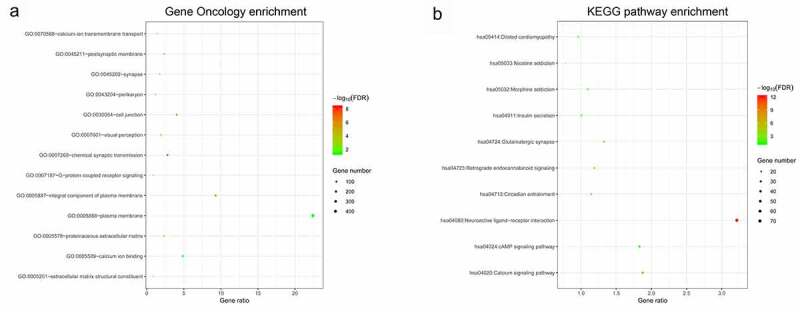
Functional enrichment analysis of lncRNA co-expression genes
Gene Oncology (GO) enrichmentKEGG pathway enrichment.

In this study, 16 lncRNAs were related with CpG sites in the methylation-based classifier (Table 2). The MEM analysis was performed to identify a total of 2241 co-expressed genes of lncRNAs. The DAVID was applied to perform functional analysis of the co-expression genes. The results indicated that Gene Oncology (GO) terms were enriched conspicuously, which were related to G-protein coupled receptor signaling (biological process), cell junction (cellular component), and calcium ion binding (molecular function). KEGG analysis showed that these co-expression genes were enriched in the calcium signaling pathway and cAMP signaling pathway (Figure 6).

## Discussion

In this study, a prognostic methylation model of lncRNAs was identified from the whole genome in patients with PCa. Combined methylation analyses and LASSO Cox regression has found that the classifier based on aberrantly methylated lncRNAs conferred an OS advantage in PCa.

Although little protein potentially coded, it was indicated that some lncRNAs played crucial functions in multitudinous tumor physiological processes including regulation of cell-cyle, proliferation, differentiation, tumorigenesis as well as RNA processing and modification, nuclear remodeling, and cytoplasmic or nuclear trafficking [16]. Methylation was involved in epigenetic silencing of cancer-relevant genes including individual non-coding RNAs [17]. In this study, the CpG-rich regions located in lncRNAs were displayed in PCa patients with various survival rates, which mediated the loss-of-function of genes about 10 folds more common than by point mutations. Methylated aberrations of lncRNAs can specifically influence various biological activities or pathways. The resulting functional annotation showed that the methylated aberration of lncRNAs can perturb tumor-related signal pathways, containing the regulation of transcription, cell differentiation, protein kinase activation, nucleotide binding, cellular target components, RAS/RAF/MEK/MAPK, and PI3K/Akt /mTOR signaling pathways [18]. PI3Ks were rich in intracellular membranes and can induce phosphatidylinositol 3,4,5-trisphosphate production [19]. AKT serine/threonine kinases can be phosphorylated by phospholipids in a PI3K-dependent manner to activate numerous substrates, thereby promoting cellular growth, division cycle, and progression.

The well-documented function of methylated lncRNAs suggested that methylated lncRNAs can influence the DNA-specific binding domain and regulate various signal transductions including G-protein coupled receptor signaling, cAMP, cell junction, and Calcium [20]. Activation of G-protein-coupled signaling associated with upregulated downstream signaling components has been shown in many tumors [21]. The dysregulation of cAMP signaling has proven to be implicated in the pathophysiological disorders of cancer and can be utilized as a prospective preventive strategy for antitumor intervention [22]. Ca2+-second-messenger plays key roles in the activation or repression of many signal transductions. The dysregulation of Ca 2+ homeostasis leads to tumorigenesis, such as gene transcription, angiogenesis, proliferation, and cellular apoptosis, and several tumors are closely associated with Ca (2+) signals [23].

The prognosis resulting from patients with advanced PCa was very grave, the median duration was shorter in patients with clinically detectable metastases. Once there is metastasis, there are limited therapeutic interventions for PCa. Thus, early diagnosis of PCa is important to reduce mortality in patients, which requires reliable prognosticators [24]. In our study, we applied Cox-regression techniques with LASSO to prognostically classify all patients, and based upon their own quantified methylation levels in 16 sites of lncRNAs, respectively. The PCa was subsequently divided into groups of higher and lower risk by median risk scores, indicating that a worse prognosis appeared in the high-risk subgroup rather than in the low-risk subgroup. The (ROC) analysis indicated that our prognostic model had optimal performance in predicting the overall 3,5 and 7-year survival of PCa.

Previous data have shown that various lncRNAs are differentially expressed in the progressed PCa. Aberrantly methylated DNA of cancer-relevant genes has been found to be correlated with the suppression in multiple cancer cellular types [25]. Therefore, based upon the combined methylation panel of lncRNAs, we integrate aberrant methylation of lncRNAs into prognostic assessments using the LASSO-Cox regression tool with a high level of prognosis accuracy. The biological function of lncRNAs used for our classifier has been shown to be associated with other cancers in multiple previous studies. The site cg02893550 in the gene symbol of the lncRNA-CpG gene is FGF14-AS2, and the correlation between FGF14-AS2 and miR-205-5p was validated in breast cancer cells. Overexpression of FGF14-AS2 impaired the miR-205-5p induced phenotypic characteristics on proliferation, invasion, migration, and apoptosis in breast cancer cells [26]. It is identified that LINC01122 (cg09671962) is closely associated with OS coupled with a high risk of gastric cancer [27]. It was proved that MEG3 (cg14245102) can affect Wnt-β-catenin pathway and control epithelial-mesenchymal transition [28]. As defined for lncRNA, the upregulation of PVT1 (cg24514600) is proved to promote cancer cell proliferation, invasion, metastasis, and drug resistance [29].

The limitations in the current research can be further studied in the future. First, the results of prognosis based upon methylated panel analysis were obtained from the PCa database of TCGA, so the predictive panel accuracy should be tested in an alternative clinical cohort. Secondly, the sample size was limited in our study. Considering the incidence of PCa, the sample sizes need to be expanded in varied regions. Thirdly, the tissues of PCa patients were used in our research, while the blood samples are much easily obtained in the clinic. Thus, further prospective studies including groups in varied regions with consistently applied protocols would be needed to validate and confirm these biomarker candidates in the near future.

## Conclusion

In summary, the differential methylation sites of lncRNAs between PCa and para-carcinoma tissues were screened out. Among these aberrant methylation sites of lncRNAs, a classifier of CpG sites in lncRNAs was established to predict the OS. Furthermore, the methylation-based classifier had admirable prediction performance, which could offer a novel insight for screening prognosis biomarkers of PCa.

## Data Availability

The DNA methylation data, RNA-seq data and clinical information of PCa patients were obtained from TCGA dataset (https://portal.gdc.cancer.gov/). The DNA methylation profile was performed by Infinium HumanMethylation450 BeadChip, The RNA-seq data was performed on the IlluminaHiSeq RNA-seq platform. The annotation files of lncRNAs were obtained from GENCODE (https://www.gencodegenes.org/). All datasets were publicly available and the study met the publication guidelines.

## Highlights

1. Differential methylation sites of lncRNAs between PCa and para-carcinoma tissues were screened out.
2. Sixteen CpG sites were selected by LASSO Regression and a risk score formula for methylated-based classifier was established.
3. The time-dependent ROC was used to evaluate the precision of the classifier for predicting the OS.
